# Diagnostics and treatment delay in primary central nervous system lymphoma: What the neurosurgeon should know

**DOI:** 10.1007/s00701-024-06138-3

**Published:** 2024-06-11

**Authors:** M. C. Hasner, M. P. van Opijnen, M. van der Meulen, R. M. Verdijk, S. L. N. Maas, L. C. J. te Boome, M. L. D. Broekman

**Affiliations:** 1 Department of Neurosurgery, Haaglanden Medical Centre, The Hague, The Netherlands; 2 Department of Neurosurgery, Leiden University Medical Centre, Leiden, The Netherlands; 3 Department of Neurology, Medisch Spectrum Twente, Enschede, The Netherlands; 4 Department of Pathology, Leiden University Medical Centre, Leiden, The Netherlands; 5 Department of Pathology, Erasmus MC Cancer Institute, University Medical Center Rotterdam, Rotterdam, The Netherlands; 6 Department of Hematology, Haaglanden Medical Centre, The Hague, The Netherlands

**Keywords:** Primary central nervous system lymphoma, Stereotactic biopsy, Cerebrospinal fluid-based diagnostics, Diagnostic delay

## Abstract

**Purpose:**

The gold standard for diagnostics in primary central nervous system lymphoma (PCNSL) is histopathological diagnosis after stereotactic biopsy. Yet, PCNSL has a multidisciplinary diagnostic work up, which associated with diagnostic delay and could result in treatment delay. This article offers recommendations to neurosurgeons involved in clinical decision-making regarding (novel) diagnostics and care for patients with PCNSL with the aim to improve uniformity and timeliness of the diagnostic process for patients with PCNSL.

**Methods:**

We present a mini review to discuss the role of stereotactic biopsy in the context of novel developments in diagnostics for PCNSL, as well as the role for cytoreductive surgery.

**Results:**

Cerebrospinal fluid-based diagnostics are supplementary and cannot replace stereotactic biopsy-based diagnostics.

**Conclusion:**

Histopathological diagnosis after stereotactic biopsy of the brain remains the gold standard for diagnosis. Additional diagnostics should not be a cause of diagnostic delay. There is currently no sufficient evidence supporting cytoreductive surgery in PCNSL, with recent studies showing contradictive data and suboptimal study designs.

## Introduction

Primary central nervous system lymphomas are rare extranodal non-Hodgkin lymphomas confined to the central nervous system, without systemic involvement. According to the WHO classification of Central Nervous System Tumours 5th edition, four more common types can be defined: primary large B-cell lymphoma of the CNS (PCNS-LBCL), immunodeficiency-associated CNS lymphomas, lymphomatoid granulomatosis and intravascular large B-cell lymphoma [42]. Miscellaneous rare lymphomas that can primarily occur in the CNS are MALT lymphoma of the dura, other low-grade B-cell lymphomas of the CNS, anaplastic large cell lymphoma (ALK + /ALK −) and T-cell and NK/T-cell lymphomas. Of these, PCNSL-LBCL is the most common tumor type encountered and will be the focus of this review. In the more recent WHO classification of Hematolymphoid Tumours 5th edition, PCNS-LBCL is classified with the category of primary large B-cell lymphoma of immune-privileged sites, together with primary large B-cell lymphoma of the brain, spinal cord, vitreoretina, cerebrospinal fluid (CSF) and of the testis. PCNS-LBCL is otherwise known as primary CNS lymphoma (PCNSL) and shall be referred to as such in this article. PCNSL comprises less than 1% of lymphomas and 4% of brain tumors [3]. PCNSL has an incidence of approximately 0.44 per 100,000, which is increased in patients > 60 years old [50, 66, 70]. The median age at presentation is 65 years old [47]. In cases of immunodeficiency, autoimmune disease, or immunosuppressive medication, PCNSL is more prevalent and is typically Eppstein-Barr virus (EBV) related. Presenting symptoms include cognitive and behavioral changes, focal deficits, headache, visual complaints, and/or epilepsy, depending on the localization of PCNSL [40]. 25–40% of PCNSL patients present with leptomeningeal disease [63].

Untreated PCNSL has a disconcerting overall survival of 1.5 months [31]. High-dose methotrexate is the cornerstone of treatment for PCNSL, but there is currently no international uniform treatment for PCNSL [58]. In population-based studies, the median survival time of PCNSL is 16 months [50]. However, the prognosis is age dependent and worsens at higher age. PCNSL patients undergoing treatment, have a 5-year overall survival (OS) in the total population of 35% and a 5-year OS in elderly (i.e. > 70 years-old) of 6% [66]. In recent trials, 2- and 3-year OS was 70–80%, and 60%, respectively [10, 22]. Predictive models using prognostic parameters were developed for clinical care and the design of prospective studies. The Memorial Sloan Kettering Cancer Centre uses > 50 years and a Karnofsky Performance Status (KPS) score of < 70 as factors predicting a worse prognosis [2]. The International Extranodal Lymphoma Study Group model considers an age > 60 years, Eastern Cooperative Oncology Group (ECOG) clinical performance > 1, increased serum lactate dehydrogenase (LDH), elevated protein in cerebrospinal fluid, and involvement of deep brain structures (i.e. periventricular, brain stem, basal ganglia, and/or cerebellum) as parameters for a negative outcome [20]. However, the latter model has not been externally validated and experts warn against the use of KPS as a prognostic marker in PCNSL, due to possible rapid neurological deterrence at onset of disease. Comorbidity and performance prior to onset of disease are of importance in an elderly population.

The gold standard for diagnosis of PCNSL is histopathological diagnosis after stereotactic biopsy [4]. This article outlines the diagnostic work-up for suspected PCNSL and aims to provide neurosurgeons a summary of the current treatment options and prognostic factors of PCNSL.

## Diagnostic work-up

### Neuroimaging

**Fig. 1:**
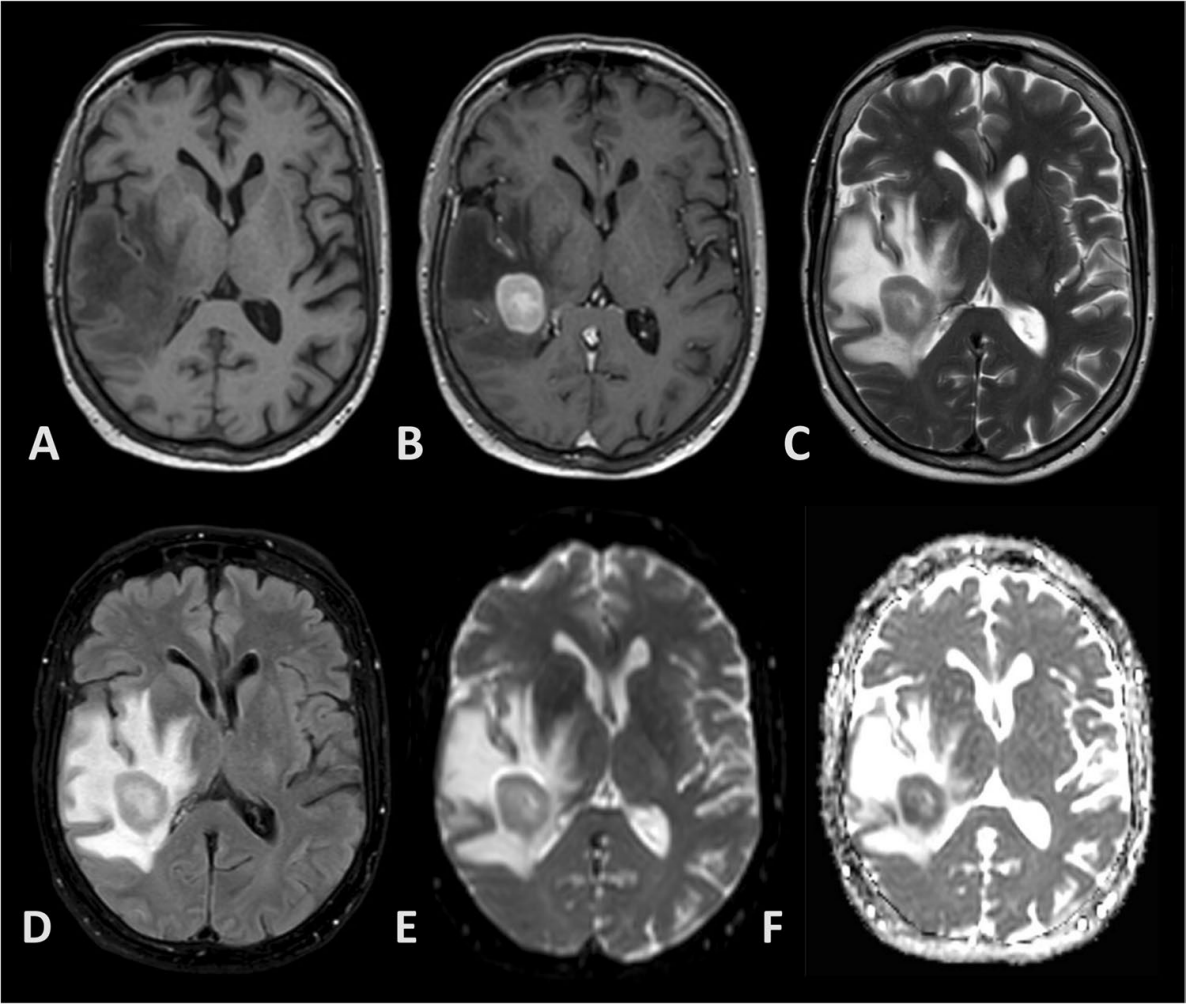
Example of magnetic resonance imaging (MRI) of the brain for patient suffering from primary central nervous system lymphoma (PCNSL). **a** T1-weighted; **b** T1-weighted post gadolinium; **c** T2-weighted; **d** Fluid-attenuated inversion recovery (FLAIR); **e** Diffusion-weighted imaging (DWI); **f** Apparent diffusion coefficient (ADC)

On magnetic resonance imaging (MRI), lymphoma typically shows as a solitary (in circa 65% of the cases) or multiple space occupying lesions that are sharply demarcated and surrounded by vasogenic edema. Most lesions are isointense or hypointense on T2-weighted fluid-attenuated inversion recovery (FLAIR) MRI. T1-weighted imaging after contrast administration is likely to show homogenous enhancement. Over 80% of the lesions are located supratentorially with a high affinity for the periventricular regions, basal ganglia and corpus callosum [54]. Diffusion weighted imaging (DWI) and apparent diffusion coefficient (ADC) may show diffusion restriction in 90% of the patients, concordant to the high cellularity of the tumor (Fig. 1). In comparison to other intracranial tumors, PCNSL show relatively low perfusion and is unlikely to contain calcifications, bleeding, or necrosis prior to treatment. Cystic and ring-shaped enhancements are unlikely to be found, except in immunocompromised patients like human immunodeficiency virus (HIV)-related lymphoma. In rapidly expanding lesions, there could be non-homogenous enhancement on T1 + gadolinium MRI, even in immunocompetent patients [65].

Next, it is important to differentiate between primary and secondary CNSL. Secondary CNSL is based on dissemination of systemic lymphoma to the central nervous system, which is reported in 4–13% of CNSL cases [1]. Computed tomography (CT) of the neck, thorax and abdomen may reveal lymphadenopathy or other tumors eligible for biopsy, and may avert the need for stereotactic biopsy of the brain. In comparison to conventional CT imaging, positron emission tomography (PET) imaging can prove more sensitive in identifying systemic illness and is preferred [45]. However, to avoid diagnostic delay, subsequent imaging with PET-CT can be performed after histopathological confirmation of PCNSL.

### Stereotactic biopsy versus vitreous- and CSF-based diagnostics

**Fig. 2:**
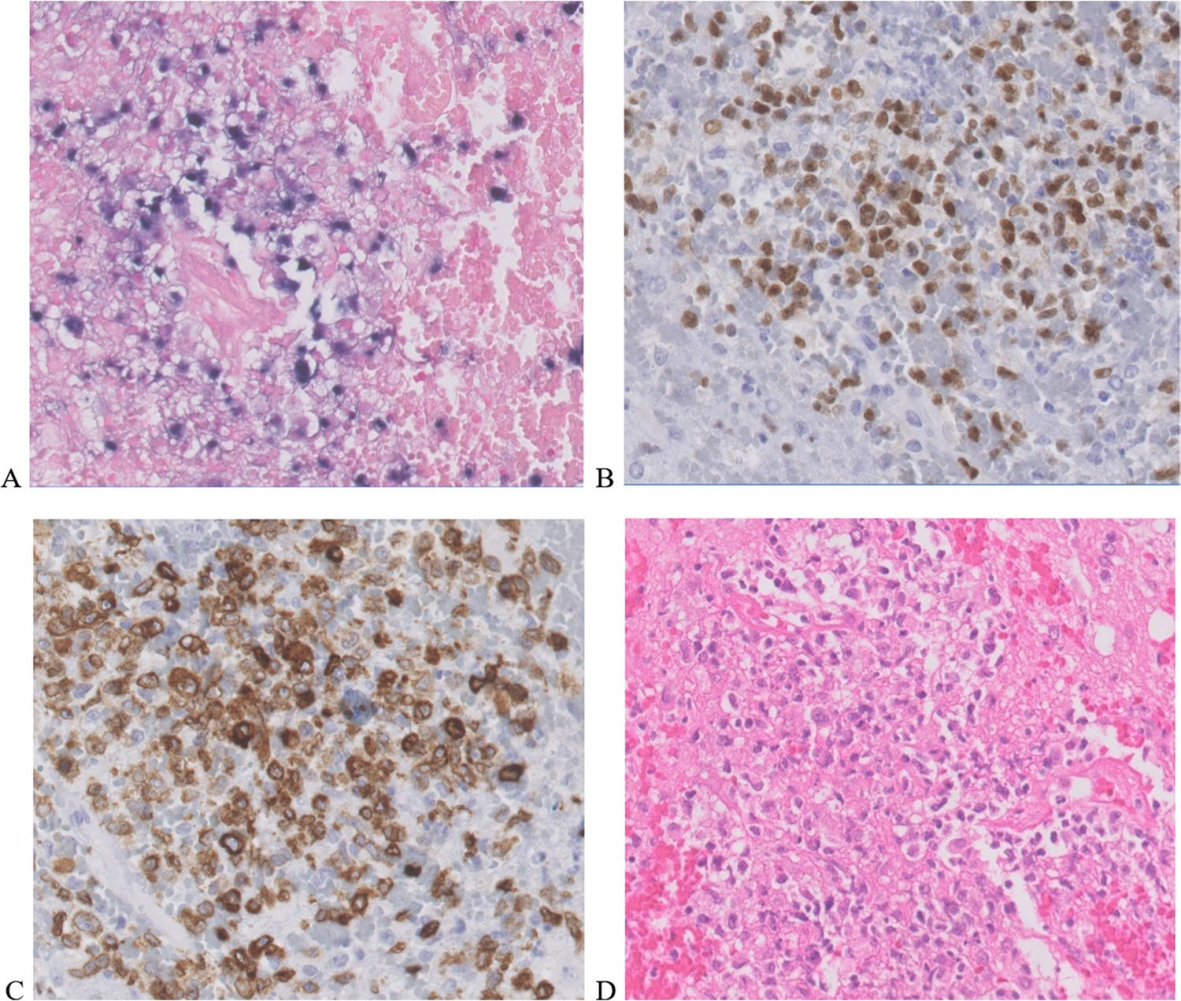
Example of microscopy images (400 × magnification) of Eppstein Bar virus-positive primary central nervous system lymphoma (PCNSL). **a** Chromogen situ hybridization (CISH) portraying a blue positive EBER stain in; **b** Immunohistochemical staining of B-cell nuclei with Pax 5; **c** Immunohistochemical staining of membrane and cytoplasm of B-cells with CD79a; **d** Histological staining with hematoxylin and eosin

The gold standard for diagnosis of PCNSL is histopathological analysis of a brain biopsy, with a diagnostic yield of over 91% [33, 60]. General considerations for neurosurgical intervention are tumor location, suspected entity and patient preferences. Other factors of influence are the prognostic factors conjointly determining eligibility for treatment, such as age, HIV-status, and clinical performance. Decision making requires interdisciplinary communication about treatment options. Following biopsy, histopathology of lymphoma typically shows infiltration of neoplastic large B-cells in the brain parenchyma with prominent nuclei, necrosis and activation of astrocytes and microglial cells. Immunohistochemical phenotyping can confirm post germinal center B-cell phenotype (MUM1 + ; BCL6 + ; CD10-) to exclude alternative diagnoses [35]. Chromogen in situ hybridization (CISH) can be used to detect EBV-encoded ribonucleic acids (RNAs), such as EBER (Fig. 2), to exclude EBV driven large B-cell lymphoma (LBCL). EBV-driven immunodeficiency-associated CNS lymphomas are associated with a worse prognosis [55]. In low cellularity biopsies or biopsies taken after corticosteroid treatment, molecular *MYD88* testing or B-cell clonality analysis of immunoglobulin genes can be performed [11]. When a B-cell clone is detected, this enables the differentiation between PCNSL from reactive inflammatory lesions or “vanished lymphoma” cases, subsequent to administration of corticosteroids. Furthermore, demonstration of *MYD88*, *CD79b* or other mutations may offer additional diagnostic value in cases where histology is not definitive, as for example *MYD88* is mutated in approximately 85% of PCNSL [33]. The combination of these tests may support diagnosis with a high sensitivity and specificity for PCNSL[5, 74], but neither are required for confirmation of diagnosis [23].

Preferably, all patients should undergo a lumbar puncture (LP) for cytopathology and immunophenotyping by flowcytometry of CSF for tumor staging. In any case, CSF diagnostics should not delay stereotactic biopsy for diagnostic purposes. CSF cytopathology reveals an increase in large blast-like lymphocytes in CSF in 25–40% of patients with PCNSL. Immunophenotyping can further distinguish B-cell from T-cell lymphoma with a combination of cluster of differentiation (CD) markers either by immunocytopathologic or flowcytometric means. Negative cytopathology and flowcytometric immunophenotyping do not rule out PCNSL, because these methods have a low sensitivity of 2–32% [61]. Clonality analysis of immunoglobulin genes and PCR testing of *MYD88* hotspot mutations on CSF may again be of additional diagnostic value. When stereotactic biopsy is not feasible, diagnosis can be made based on a combination of typical presentation on MRI in combination with immunophenotyping of CSF with the potential addition of molecular makers. If the diagnosis remains uncertain, biopsy is inevitable. Furthermore, in patients known with a systemic lymphoma in the recent medical history, an LP is sufficient to diagnose CNS involvement. This is supported by the notion that secondary CNS lymphoma more often present with leptomeningeal disease, rather than intraparenchymal [25, 34].

In exceptional cases where stereotactic biopsy and LP are not feasible, vitreous biopsy could confirm the diagnosis [17]. In patients with vitreoretinal lymphoma (VRL) diagnostic vitrectomy has a sensitivity of 77% [41]. However, ocular dissemination is present at diagnosis in 10–25% of patients with PCNSL [19]. All patients should be referred to the ophthalmologist for tumor staging [35]. Intra-ocular localization could have consequences for treatment administration and follow-up [28]. Vitreous biopsy could take place if intra-ocular examination shows cellular infiltration, or subretinal tumor cell infiltrates are observed on fundoscopy. For vitreous samples the same set of tests described for CNS sampling can be performed with the addition of IL10/IL6 cytokine testing, and excluding the differential diagnosis of EBV-driven PCNSL remains of importance [62].

Evidently, the question arises whether brain biopsy is indicated if a sampling of CSF or vitreous fluid can be performed. Literature has suggested that identification of lymphoma cells in CSF or vitreous fluid in combination with clinical and radiological features of PCNSL are confirmative for PCNSL and may avert the need for (high risk) biopsy [35]. Unfortunately for diagnostic purposes, the incidence of ocular dissemination is low and prioritizing ocular biopsy could result in diagnostic delay. In only 7% of patients with clinical and radiological features suspect for PCNSL diagnosis can be made upon LP [29, 46]. In addition to the low sensitivity of LP-based cytopathology and immunophenotyping of CSF. Unfortunately, diagnosis cannot be made solely on cytopathology. Histopathology is needed to examine the morphology, hemotoxylin and eosin (H&E) staining, immunohistochemistry (IHC), B-cell immunophenotype analysis and testing for EBV-driven RNAs. Hematologists suggest that a LP is not sufficient for the extensive molecular and immunophenotype analysis necessary for exact WHO tumor classification and determination of treatment [17].

Consequently, it is advised to perform a stereotactic biopsy of the brain as soon as possible. Histopathological diagnosis obtained after stereotactic biopsy remains the gold standard for diagnosing a PCNSL and should not be delayed to perform CSF diagnostics. Clinicians involved should strive to minimize delay in the diagnostic process of PCNSL to prevent progressive neurological symptomatology. Old age, clinical performance and treatment delay are correlated with a reduced survival in patients with PCNSL, who already have a limited prognosis [2, 12].

### Following histological confirmation of CNSL

After histological determination that the lesion is a lymphoma, the patient is referred to the hematologist for tumor staging. Subsequent imaging of the neck, thorax, and abdomen with PET-CT should be performed to differentiate between primary and secondary CNSL, and for tumor staging. In 10% of CNSL cases it concerns SCNSL, oftentimes based on recurrence of systemic lymphoma, or discovery of an intravascular or testicular secondary localization. Therefore, physical examination (and potentially ultrasound) of the testis should be performed, as well as additional blood tests, including blood count, immunoglobulins (IgM, IgG and IgA), LDH, kidney- and liver function, and the left ventricular ejection fraction (LVEF) of cardiovascular patients. Furthermore, all patients presenting with PCNSL should be tested for Hepatitis B, C, and additionally HIV and Quantiferon in high-risk populations [35]. Bone marrow diagnostics have no additional value for tumor staging [29].

### PCNSL diagnostics and corticosteroids

Patients with a symptomatic intracranial mass are often prescribed corticosteroids to ameliorate symptoms and reduce cerebral edema. It is crucial that clinicians treating patients with PCNSL prior to histopathological diagnosis refrain from prescribing corticosteroids if possible [59]. Dexamethasone induces apoptosis of PCNSL cells and could lead to temporary disappearance, otherwise known as “vanished lymphoma”, which complicates histopathological diagnostics due to morphological changes and transient tumor mass reduction [27, 49]. The administration of corticosteroids is associated with inconclusive biopsy by an odds ratio of 3.3 [59]. Even a single dose of corticosteroids could lead to inconclusive results [43]. In case corticosteroids have been given, it is recommended to postpone (re-)biopsy until serial MRI imaging indicates new tumor growth [35]. Alternatively, in patients with symptomatic lesions or edema suspected for lymphoma, osmotic treatment with mannitol or hypertonic saline could be considered to lower intracranial pressure [57]. Of note, vanishing of the enhancement under corticosteroid administration is not specific nor diagnostic for lymphoma [8].

After biopsy, treatment with dexamethasone can be started to reduce intracranial edema and the consequential neurological symptoms. There is no consensus about dosage and duration of treatment. There is no evidence suggesting that dexamethasone leads to prolonged survival in patients with PCNSL, therefore it could be argued not to administer corticosteroids in the absence of edema-related symptoms. Long-term treatment is not indicated and especially contra-indicated in immunocompromised patients, due to the possible side-effects of glucocorticoids and the inevitable relapse of lymphoma [57].

### Role for cytoreductive surgery

Neurosurgical intervention is usually limited to stereotactic biopsy for pathological diagnosis. PCNSL often presents with multiple, diffuse deep-seated lesions. In the past, resection has been advised against, because of the risk of developing surgery-related neurological deficits and the high sensitivity of PCNSL to chemotherapy, whole brain radiotherapy (WBRT), and autologous stem cell transplantation (ASCT) [21, 24, 37]. The recommendation against resection was based on small retrospective studies failing to show cytoreductive surgery has a benefit over supportive care or over biopsy followed by chemo- and radiotherapy [6, 7, 15, 32]. However, this data may no longer reflect modern neurosurgery with novel imaging- and navigation techniques.

Recently, larger series have shown the possible benefits of resection for PCNSL [56, 73]. A retrospective analysis of a phase 3 trial comprising 526 patients showed that 67 patients undergoing (sub)total resection had a significantly better progression free survival (PFS) and overall survival (OS) in comparison to patients undergoing biopsy, independently of age and clinical performance. [73] After adjusting for patients with single lesions more often undergoing resection, only PFS remained statistically significant. There is a strong risk of bias though, given the study does not adjust for localization of the tumor in surgically inaccessible areas of the brain, which has a negative impact on both prognosis and the decision to perform resection. A retrospective case control study of the National Cancer Database (NCDB) found a median survival (MS) of 19.5 versus 11.0 months in 8936 patients undergoing craniotomy versus biopsy respectively, independent of subsequent treatment with chemo-/radiotherapy [56]. The benefit of craniotomy over biopsy increased after adjusting for age with a MS of 95.1 versus 29.1 months respectively in age < 50 years (HR 0.66, p < 0.001) and 14.9 versus 10.0 months in age > 50 years (HR 0.86, p < 0.001). Benefit of craniotomy over biopsy was statistically dependent on patient risk stratification based on comorbidity, tumor focality, deep versus superficial lesions, comorbidity, performance in activities of daily living, and age. Both studies propose reevaluation of resection of PCNSL in single lesions with low-risk localization.

In contrast, a recent study found no significant difference in OS or PFS in 105 patients with single lesion PCNSL undergoing biopsy versus resection [13, 60]. Another retrospective analysis in 1002 patients of the French oculo-cerebral lymphoma network (LOC) database showed a benefit in univariate analysis of resection versus biopsy of PCNSL with an OS of 23.8 months versus 37.3 months, respectively. However, the results were not significant in multivariate analysis [38]. In conclusion, recent study designs are suboptimal, and data is contradictive. Consequently, there is no consensus on whether resection leads to prolonged survival or whether resection should take place in patients with unifocal and resectable lesions. Though resection has become safer with modern surgical and imaging techniques, it is currently not the standard of care for PCNSL and is advised only in cases with the risk of impending brain herniation or rapidly progressive neurological deterioration [7, 18].

### Treatment and follow up: what the neurosurgeon needs to know

Following neurosurgical intervention, patients are referred to the hematologist or neuro-oncologist for induction treatment with chemotherapy. It is important to start treatment for PCNSL as quickly as possible, since delay of induction treatment is associated with reduced PFS in PCNSL patients [12, 58]. Currently, there is no uniform treatment for PCNSL and there are very few controlled studies available [53]. For induction treatment the European Association of Neuro-Oncology (EANO) recommends intravenous administration of high-dose methotrexate, best administered in combination with chemotherapeutic agents with the ability to cross the blood–brain barrier [36, 76]. The chemotherapeutic drug of choice is preferably cytarabine or combinations used in randomized prospective trials, such as the alkylating agents temozolomide, procarbazine, thiotepa and carmustine [9, 21, 37]. The role of rituximab in the treatment of PCNSL is still unclear. One phase II study showed an improved PFS and OS when rituximab and thiotepa were added to HD-MTX and cytarabine [22]. However, a larger phase III study showed no benefit of adding rituximab to MBVP chemotherapy [9]. In the Netherlands patients < 70 years receive methotrexate, etoposide carmustine and predison (MBVP) and rituximab, methrotrexate, cytarabine and thiotepa (MATRix), where patients > 65 years are eligible for rituximab, methotraxeate and procarbazine (R-MP) [14, 21, 26]. Response rate is measured on MRI, and in case of CSF localization and/or intra-ocular involvement also with an LP and/or fundus examination, respectively. The induction treatment is followed by consolidation treatment with chemotherapy followed by ASCT or WBRT [22, 35, 37].

Treatment with ASCT may result in longer remission, but it is advised against in patients over 65–70 years of age or with a poor KPS due to high toxicity [22, 37]. WBRT is associated with neurotoxicity and the possible advantage of progression-free survival should therefore be weighed against the risk of toxicity in long term survivors [64]. WBRT is not recommended in patients with HIV or in those over the age of 61 years [16, 35, 71]. However, due to the limited prognosis of the disease and its correlation with neurocognitive symptoms, WBRT could nonetheless be discussed with a radiotherapist. Prognostic factors such as performance status, renal function, HIV-status and age should be taken into account conjointly to prevent undertreatment of patients that are able to tolerate more aggressive treatment [35, 67, 72]. Unfortunately, PCNSL is characterized by a high relapse rate of 36–66% [39, 75]. If there is at least a partial response (i.e. a reduction of 50% of the enhancing tumor), the extent of radiological response does not seem to influence survival rates in PCNSL [1, 68].

### Diagnostic delay and treatment delay

There are several intervals between the diagnostic procedures and the start of treatment of PCNSL where significant delay can occur. Primarily, the time span from clinical onset of symptoms to neuroimaging. A study showed this period can last up to a median 21 days in patients without HIV [30]. Factors of influence on a delay in radiological diagnostics are presenting symptoms of personality changes or visual disturbance. Neurocognitive disturbance is an unfavorable prognostic marker [69]. A study that compared the diagnostic delay in patients with PCNSL and patients with glioblastoma found a median delay of 19 days and 9.5 days respectively, suggesting that patients with PCNSL are more at risk of a delayed neurosurgical intervention. The delay was not in the period from clinical onset of symptoms to imaging, but seemed to occur between neuroimaging and morphological diagnosis [12]. Literature suggests that early biopsy for PCNSL is safe, valid, and may minimize diagnostic delay [46]. It generally takes two more weeks after biopsy until histopathological diagnosis of PCNSL and induction of treatment [48]. This results in a cumulative delay of more than 30 days, which is strongly correlated with a reduced survival compared to induction of treatment within one month [12].

## Discussion

Given the high specificity and sensitivity of brain biopsy, this remains the gold standard for diagnosis of PCNSL. Cytoreductive surgery remains advised against, due to the risk of developing neurological deficits subsequent to resection and the high sensitivity of PCNSL to chemotherapy, ASCT and WBRT. This recommendation is based on small retrospective studies in the past and their data may no longer reflect modern neurosurgery, guided by novel imaging- and navigation techniques. Recent literature is contradictive, though there are several studies suggesting a benefit in survival related to resection over biopsy in PCNSL. It would be interesting to explore in a prospective cohort study whether resection of superficial, unifocal lesions in non-eloquent regions leads to better overall survival in comparison to biopsy in patients with PCNSL.

Various fields of medicine are involved in the diagnostic work up of PCNSL. Stereotactic biopsy should be prioritized and should not be delayed by CSF diagnostics or vitreous biopsy, as the gold standard remains histopathological diagnosis. CSF diagnostics does not provide enough material for sufficient testing to obtain diagnosis, as it does not provide the tissue that is per definition needed for histological diagnosis. Similarly, vitreous biopsy provides material for cytology, but does not allow for the same diagnostic certainty as brain biopsy. In conclusion, CSF diagnostics and vitreous biopsy are no realistic substitutes for brain biopsy due to their low sensitivity and the risk of reaching no pathological confirmation of diagnosis. However, when brain biopsy is not feasible, these alternatives should be considered. Immunocytopathology is necessary to confirm PCNSL. Molecular clonality analysis of immunoglobulin genes or mutation in specific genes (e.g. *MYD88*, *CD79b*) may further support the diagnosis, but they are optional and not standard practice. Administration of corticosteroids should be postponed until a brain biopsy has been performed, because it is associated with inconclusive biopsy and a delay in treatment. After histological confirmation of CNSL, subsequent diagnostics should differentiate between primary and secondary CNSL.

Diagnostic delay of PCNSL is correlated with treatment delay, which is associated with earlier relapse and reduced survival in PCNSL [12, 58]. Treatment selection is influenced by prognostic markers, such as age, clinical performance, and HIV-status. Upon progression of disease, clinical performance may deteriorate due to progression of neurocognitive symptomology. Evidently, some causes of delay are influenced by the organization of care, thus are subordinate to hospital structures and contextual factors. Retrospective research compared treatment selection for PCNSL patients in a public safety-net hospital to a private tertiary academic institution in America [52]. It was suggested that sociodemographic barriers impact treatment selection and its outcome [51].

As treatment options for the elderly population (> 70 years) are being developed, the requests for stereotactic biopsy in vulnerable patients may increase. Clinicians should be aware of the vulnerability of the elderly population with PCNSL. They are particularly at risk of worsening their already unfavorable prognosis, when they suffer from factors associated with delayed diagnostics, such as vision loss or neurocognitive problems.[30] As the median OS of patients with PCNSL under the age of 70 doubled over a 40-year period to 2010, the survival of elderly patients remained unchanged with a marginal 6–7 months [44]. Fields of medicine involved in the care for patients with PCNSL should strive to determine and optimize factors influencing timely diagnostics and induction of treatment. Therefore, patients suspect of PCNSL should preferably be referred to a clinical setting where all diagnostic and treatment modalities are available to avoid delay.

## Data Availability

Not applicable.

## References

[1] Abrey LE, Batchelor TT, Ferreri AJ, Gospodarowicz M, Pulczynski EJ, Zucca E et al (2005) Report of an international workshop to standardize baseline evaluation and response criteria for primary CNS lymphoma. J Clin Oncol 23(22):5034–5043 15955902 10.1200/JCO.2005.13.524

[2] Abrey LE, Ben-Porat L, Panageas KS, Yahalom J, Berkey B, Curran W et al (2006) Primary central nervous system lymphoma: the Memorial Sloan-Kettering Cancer Center prognostic model. J Clin Oncol 24(36):5711–5715 17116938 10.1200/JCO.2006.08.2941

[3] Alaggio R, Amador C, Anagnostopoulos I, Attygalle AD, Araujo IBO, Berti E et al (2022) The 5th edition of the world health organization classification of haematolymphoid tumours: Lymphoid neoplasms. Leukemia 36(7):1720–48 35732829 10.1038/s41375-022-01620-2 PMC9214472

[4] Baraniskin A, Schroers R (2021) liquid biopsy and other non-invasive diagnostic measures in PCNSL. Cancers (Basel) 13(11) 10.3390/cancers13112665 PMC8198992 34071407

[5] Baraniskin A, Zaslavska E, Nöpel-Dünnebacke S, Ahle G, Seidel S, Schlegel U et al (2016) Circulating U2 small nuclear RNA fragments as a novel diagnostic biomarker for primary central nervous system lymphoma. Neuro Oncol 18(3):361–367 26250566 10.1093/neuonc/nov144 PMC4767235

[6] Bataille B, Delwail V, Menet E, Vandermarcq P, Ingrand P, Wager M et al (2000) Primary intracerebral malignant lymphoma: report of 248 cases. J Neurosurg 92(2):261–266 10659013 10.3171/jns.2000.92.2.0261

[7] Bellinzona M, Roser F, Ostertag H, Gaab RM, Saini M (2005) Surgical removal of primary central nervous system lymphomas (PCNSL) presenting as space occupying lesions: a series of 33 cases. Eur J Surg Oncol 31(1):100–105 15642434 10.1016/j.ejso.2004.10.002

[8] Bromberg JE, Siemers MD, Taphoorn MJ (2002) Is a “vanishing tumor” always a lymphoma? Neurology 59(5):762–764 12221174 10.1212/wnl.59.5.762

[9] Bromberg JEC, Issa S, Bakunina K, Minnema MC, Seute T, Durian M et al (2019) Rituximab in patients with primary CNS lymphoma (HOVON 105/ALLG NHL 24): a randomised, open-label, phase 3 intergroup study. Lancet Oncol 20(2):216–228 30630772 10.1016/S1470-2045(18)30747-2

[10] Bromberg JEC, Issa S, van der Holt B, van der Meulen M, Dirven L, Minnema MC et al (2023) Survival, neurocognitive function, and health-related quality of life outcomes after rituximab—methotrexate, BCNU, teniposide, and prednisolone for primary CNS lymphoma: Final results of the HOVON 105/ALLG NHL 24 study. Neuro Oncol 26(4):724–734 10.1093/neuonc/noad224 PMC10995504 38037691

[11] Brück W, Brunn A, Klapper W, Kuhlmann T, Metz I, Paulus W et al (2013) Differential diagnosis of lymphoid infiltrates in the central nervous system: experience of the network lymphomas and lymphomatoid lesions in the nervous system. Pathologe 34(3):186–197 23471726 10.1007/s00292-013-1742-9

[12] Cerqua R, Balestrini S, Perozzi C, Cameriere V, Renzi S, Lagalla G et al (2016) Diagnostic delay and prognosis in primary central nervous system lymphoma compared with glioblastoma multiforme. Neurol Sci 37(1):23–29 26233232 10.1007/s10072-015-2353-4

[13] Cheng X, Chen H, Sun C, Zhang B, Zhang J, Wang Y (2022) Whether surgical resection or biopsy makes difference in single lesion primary central nervous system lymphoma: a single center retrospective cohort study. BMC Neurol 22(1):411 36333683 10.1186/s12883-022-02930-9 PMC9636826

[14] Colombat P, Lemevel A, Bertrand P, Delwail V, Rachieru P, Brion A et al (2006) High-dose chemotherapy with autologous stem cell transplantation as first-line therapy for primary CNS lymphoma in patients younger than 60 years: a multicenter phase II study of the GOELAMS group. Bone Marrow Transplant 38(6):417–420 16951691 10.1038/sj.bmt.1705452

[15] DeAngelis LM, Yahalom J, Heinemann MH, Cirrincione C, Thaler HT, Krol G (1990) Primary CNS lymphoma: combined treatment with chemotherapy and radiotherapy. Neurology 40(1):80–86 2296388 10.1212/wnl.40.1.80

[16] Doolittle ND, Korfel A, Lubow MA, Schorb E, Schlegel U, Rogowski S et al (2013) Long-term cognitive function, neuroimaging, and quality of life in primary CNS lymphoma. Neurology 81(1):84–92 23685932 10.1212/WNL.0b013e318297eeba PMC3770201

[17] Doorduijn JKLtB, Durian M, Klerk C, Minnema M, Nijland M, Oostvogels R, van der Poel MWM, Vermaat JSP, Zijlstra J, Anten M, Bromberg J, Fonville S, van der Meulen M, Ta B, Abdul Hamid M, Jansen PM, de Vries TS (2024) Richtlijnen: Primair zenuwstelsel lymfoom PCZSL [updated 30–1–24. Available from: https://publicatie.hematologienederland.nl/richtlijnen/primair-zenuwstelsel-lymfoom-pczsl/

[18] Elder JB, Chen TC (2006) Surgical interventions for primary central nervous system lymphoma. Neurosurg Focus 21(5):E13 17134115 10.3171/foc.2006.21.5.14

[19] Eloranta S, Brånvall E, Celsing F, Papworth K, Ljungqvist M, Enblad G et al (2018) Increasing incidence of primary central nervous system lymphoma but no improvement in survival in Sweden 2000–2013. Eur J Haematol 100(1):61–68 28983970 10.1111/ejh.12980

[20] Ferreri AJ, Blay JY, Reni M, Pasini F, Spina M, Ambrosetti A et al (2003) Prognostic scoring system for primary CNS lymphomas: the International Extranodal Lymphoma Study Group experience. J Clin Oncol 21(2):266–272 12525518 10.1200/JCO.2003.09.139

[21] Ferreri AJ, Cwynarski K, Pulczynski E, Ponzoni M, Deckert M, Politi LS et al (2016) Chemoimmunotherapy with methotrexate, cytarabine, thiotepa, and rituximab (MATRix regimen) in patients with primary CNS lymphoma: results of the first randomisation of the International Extranodal Lymphoma Study Group-32 (IELSG32) phase 2 trial. Lancet Haematol 3(5):e217–e227 27132696 10.1016/S2352-3026(16)00036-3

[22] Ferreri AJM, Cwynarski K, Pulczynski E, Fox CP, Schorb E, La Rosée P et al (2017) Whole-brain radiotherapy or autologous stem-cell transplantation as consolidation strategies after high-dose methotrexate-based chemoimmunotherapy in patients with primary CNS lymphoma: results of the second randomisation of the International Extranodal Lymphoma Study Group-32 phase 2 trial. Lancet Haematol 4(11):e510–e523 29054815 10.1016/S2352-3026(17)30174-6

[23] Ferreri AJM, Calimeri T, Lopedote P, Francaviglia I, Daverio R, Iacona C et al (2021) MYD88 L265P mutation and interleukin-10 detection in cerebrospinal fluid are highly specific discriminating markers in patients with primary central nervous system lymphoma: results from a prospective study. Br J Haematol 193(3):497–505 33620087 10.1111/bjh.17357

[24] Ferreri AJM, Cwynarski K, Pulczynski E, Fox CP, Schorb E, Celico C et al (2022) Long-term efficacy, safety and neurotolerability of MATRix regimen followed by autologous transplant in primary CNS lymphoma: 7-year results of the IELSG32 randomized trial. Leukemia 36(7):1870–1878 35562406 10.1038/s41375-022-01582-5

[25] Fonti R, Salvatore B, De Renzo A, Nicolai E, Del Vecchio S (2016) Detection of Leptomeningeal Involvement by 18F-FDG-PET/CT in a Patient With Non-Hodgkin Lymphoma. Clin Nucl Med 41(2):169–172 26545028 10.1097/RLU.0000000000001060

[26] Fritsch K, Kasenda B, Schorb E, Hau P, Bloehdorn J, Möhle R et al (2017) High-dose methotrexate-based immuno-chemotherapy for elderly primary CNS lymphoma patients (PRIMAIN study). Leukemia 31(4):846–852 27843136 10.1038/leu.2016.334 PMC5383936

[27] Gametchu B (1987) Glucocorticoid receptor-like antigen in lymphoma cell membranes: correlation to cell lysis. Science 236(4800):456–461 3563523 10.1126/science.3563523

[28] Grimm SA, McCannel CA, Omuro AM, Ferreri AJ, Blay JY, Neuwelt EA et al (2008) Primary CNS lymphoma with intraocular involvement: International PCNSL collaborative group report. Neurology 71(17):1355–1360 18936428 10.1212/01.wnl.0000327672.04729.8c PMC4109164

[29] Grommes C, Rubenstein JL, DeAngelis LM, Ferreri AJM, Batchelor TT (2019) Comprehensive approach to diagnosis and treatment of newly diagnosed primary CNS lymphoma. Neuro Oncol 21(3):296–305 30418592 10.1093/neuonc/noy192 PMC6380418

[30] Haldorsen IS, Espeland A, Larsen JL, Mella O (2005) Diagnostic delay in primary central nervous system lymphoma. Acta Oncol 44(7):728–734 16227164 10.1080/02841860500256272

[31] Han CH, Batchelor TT (2017) Diagnosis and management of primary central nervous system lymphoma. Cancer 123(22):4314–4324 28950405 10.1002/cncr.30965

[32] Henry JM, Heffner RR Jr, Dillard SH, Earle KM, Davis RL (1974) Primary malignant lymphomas of the central nervous system. Cancer 34(4):1293–1302 4607602 10.1002/1097-0142(197410)34:4<1293::aid-cncr2820340441>3.0.co;2-p

[33] Hiemcke-Jiwa LS, Minnema MC, Radersma-van Loon JH, Jiwa NM, de Boer M, Leguit RJ et al (2018) The use of droplet digital PCR in liquid biopsies: A highly sensitive technique for MYD88 p.(L265P) detection in cerebrospinal fluid. Hematol Oncol. 36(2):429–35 29210102 10.1002/hon.2489

[34] Hill QA, Owen RG (2006) CNS prophylaxis in lymphoma: who to target and what therapy to use. Blood Rev 20(6):319–332 16884838 10.1016/j.blre.2006.02.001

[35] Hoang-Xuan K, Bessell E, Bromberg J, Hottinger AF, Preusser M, Rudà R et al (2015) Diagnosis and treatment of primary CNS lymphoma in immunocompetent patients: guidelines from the european association for neuro-oncology. Lancet Oncol 16(7):e322–e332 26149884 10.1016/S1470-2045(15)00076-5

[36] Hoang-Xuan K, Deckert M, Ferreri AJM, Furtner J, Gallego Perez-Larraya J, Henriksson R et al (2022) European Association of Neuro-Oncology (EANO) guidelines for treatment of primary central nervous system lymphoma (PCNSL). Neuro Oncol 25(1):37–53 10.1093/neuonc/noac196 PMC9825335 35953526

[37] Houillier C, Taillandier L, Dureau S, Lamy T, Laadhari M, Chinot O et al (2019) Radiotherapy or Autologous Stem-Cell Transplantation for Primary CNS Lymphoma in Patients 60 Years of Age and Younger: Results of the Intergroup ANOCEF-GOELAMS Randomized Phase II PRECIS Study. J Clin Oncol 37(10):823–833 30785830 10.1200/JCO.18.00306

[38] Houillier C, Soussain C, Ghesquières H, Soubeyran P, Chinot O, Taillandier L et al (2020) Management and outcome of primary CNS lymphoma in the modern era: An LOC network study. Neurology 94(10):e1027–e1039 31907289 10.1212/WNL.0000000000008900 PMC7238921

[39] Jahnke K, Thiel E, Martus P, Herrlinger U, Weller M, Fischer L et al (2006) Relapse of primary central nervous system lymphoma: clinical features, outcome and prognostic factors. J Neurooncol 80(2):159–165 16699873 10.1007/s11060-006-9165-6

[40] Korfel A, Schlegel U, Johnson DR, Kaufmann TJ, Giannini C, Hirose T (2017) Case-based review: primary central nervous system lymphoma. Neuro-Oncology Practice 4(1):46–59 31386044 10.1093/nop/npw033 PMC6656340

[41] Levasseur SD, Wittenberg LA, White VA (2013) Vitreoretinal lymphoma: A 20-year review of incidence, clinical and cytologic features, treatment, and outcomes. JAMA Ophthalmology 131(1):50–55 23307208 10.1001/jamaophthalmol.2013.569

[42] Louis DN, Perry A, Wesseling P, Brat DJ, Cree IA, Figarella-Branger D et al (2021) The 2021 WHO classification of tumors of the central nervous system: a summary. Neuro Oncol 23(8):1231–1251 34185076 10.1093/neuonc/noab106 PMC8328013

[43] Manoj N, Arivazhagan A, Mahadevan A, Bhat D, Arvinda H, Devi B et al (2014) Central nervous system lymphoma: Patterns of incidence in Indian population and effect of steroids on stereotactic biopsy yield. Neurol India 62(1):19–25 24608449 10.4103/0028-3886.128272

[44] Mendez JS, Ostrom QT, Gittleman H, Kruchko C, DeAngelis LM, Barnholtz-Sloan JS et al (2018) The elderly left behind-changes in survival trends of primary central nervous system lymphoma over the past 4 decades. Neuro Oncol 20(5):687–694 29036697 10.1093/neuonc/nox187 PMC5892148

[45] Mohile NA, Deangelis LM, Abrey LE (2008) The utility of body FDG PET in staging primary central nervous system lymphoma. Neuro Oncol 10(2):223–228 18287338 10.1215/15228517-2007-061 PMC2613825

[46] Morell AA, Shah AH, Cavallo C, Eichberg DG, Sarkiss CA, Benveniste R et al (2019) Diagnosis of primary central nervous system lymphoma: a systematic review of the utility of CSF screening and the role of early brain biopsy. Neurooncol Pract 6(6):415–423 31832211 10.1093/nop/npz015 PMC6899047

[47] Netherlands Cancer Registry (NCR), Netherlands Comprehensive Cancer Organisation (IKNL), derived via www.iknl.nl/en/ncr/ncr-data-figures [Accessed 25–11–23]

[48] Neuhauser M, Roetzer T, Oberndorfer S, Kitzwoegerer M, Payer F, Unterluggauer JJ et al (2019) Increasing use of immunotherapy and prolonged survival among younger patients with primary CNS lymphoma: a population-based study. Acta Oncol 58(7):967–976 30994047 10.1080/0284186X.2019.1599137

[49] Önder E, Arıkök AT, Önder S, Han Ü, Sorar M, Kertmen H et al (2015) Corticosteroid pre-treated primary CNS lymphoma: a detailed analysis of stereotactic biopsy findings and consideration of interobserver variability. Int J Clin Exp Pathol 8(7):7798–7808 26339344 PMC4555672

[50] Ostrom QT, Price M, Neff C, Cioffi G, Waite KA, Kruchko C et al (2022) CBTRUS statistical report: primary brain and other central nervous system tumors diagnosed in the united states in 2015–2019. Neuro Oncol 24(Suppl 5):v1–v95 36196752 10.1093/neuonc/noac202 PMC9533228

[51] Parakh S, Gan HK (2022) Primary CNS lymphoma in the real world—Opportunities for improved outcomes in different health settings. Neuro-Oncology Practice 9(3):159–160 35601965 10.1093/nop/npac028 PMC9113271

[52] Patel AM, Ali O, Kainthla R, Rizvi SM, Awan FT, Patel T et al (2022) Primary central nervous system lymphoma: a real-world comparison of therapy access and outcomes by hospital setting. Neuro-Oncology Practice 9(3):183–192 35601974 10.1093/nop/npab066 PMC9113306

[53] Plasswilm L, Herrlinger U, Korfel A, Weller M, Küker W, Kanz L et al (2002) Primary central nervous system (CNS) lymphoma in immunocompetent patients. Ann Hematol 81(8):415–423 12223997 10.1007/s00277-002-0498-8

[54] Pons-Escoda A, Naval-Baudin P, Velasco R, Vidal N, Majós C (2023) Imaging of lymphomas involving the CNS: an update-review of the full spectrum of disease with an emphasis on the world health organization classifications of CNS Tumors 2021 and hematolymphoid tumors 2022. AJNR Am J Neuroradiol 44(4):358–366 36822829 10.3174/ajnr.A7795 PMC10084903

[55] Prado IP, Barbiero F, Baehring JM, Becker K, Corbin Z (2019) The impact of Epstein-Barr virus status on primary CNS lymphoma survival. J Clin Oncol 37(15_suppl):e13528-e

[56] Rae AI, Mehta A, Cloney M, Kinslow CJ, Wang TJC, Bhagat G et al (2019) Craniotomy and Survival for Primary Central Nervous System Lymphoma. Neurosurgery 84(4):935–944 29660011 10.1093/neuros/nyy096 PMC6500886

[57] Roth P, Wick W, Weller M (2010) Steroids in neurooncology: actions, indications, side-effects. Curr Opin Neurol 23(6):597–602 20962642 10.1097/WCO.0b013e32833e5a5d

[58] Rubenstein JL, Hsi ED, Johnson JL, Jung SH, Nakashima MO, Grant B et al (2013) Intensive chemotherapy and immunotherapy in patients with newly diagnosed primary CNS lymphoma: CALGB 50202 (Alliance 50202). J Clin Oncol 31(25):3061–3068 23569323 10.1200/JCO.2012.46.9957 PMC3753699

[59] Scheichel F, Marhold F, Pinggera D, Kiesel B, Rossmann T, Popadic B et al (2021) Influence of preoperative corticosteroid treatment on rate of diagnostic surgeries in primary central nervous system lymphoma: a multicenter retrospective study. BMC Cancer 21(1):754 34187419 10.1186/s12885-021-08515-y PMC8243818

[60] Scheichel F, Pinggera D, Popadic B, Sherif C, Marhold F, Freyschlag CF (2022) An update on neurosurgical management of primary CNS lymphoma in immunocompetent patients. Front Oncol 12:884724 35515113 10.3389/fonc.2022.884724 PMC9065338

[61] Scott BJ, Douglas VC, Tihan T, Rubenstein JL, Josephson SA (2013) A systematic approach to the diagnosis of suspected central nervous system lymphoma. JAMA Neurol 70(3):311–319 23319132 10.1001/jamaneurol.2013.606 PMC4135394

[62] Song Y, Zhang W, Zhang L, Wu W, Zhang Y, Han X et al (2016) Cerebrospinal Fluid IL-10 and IL-10/IL-6 as Accurate Diagnostic Biomarkers for Primary Central Nervous System Large B-cell Lymphoma. Sci Rep 6:38671 27924864 10.1038/srep38671 PMC5141427

[63] Taylor JW, Flanagan EP, O’Neill BP, Siegal T, Omuro A, Deangelis L et al (2013) Primary leptomeningeal lymphoma: international primary cns lymphoma collaborative group report. Neurology 81(19):1690–1696 24107866 10.1212/01.wnl.0000435302.02895.f3 PMC3812109

[64] Thiel E, Korfel A, Martus P, Kanz L, Griesinger F, Rauch M et al (2010) High-dose methotrexate with or without whole brain radiotherapy for primary CNS lymphoma (G-PCNSL-SG-1): a phase 3, randomised, non-inferiority trial. Lancet Oncol 11(11):1036–1047 20970380 10.1016/S1470-2045(10)70229-1

[65] Thurnher MM, Thurnher SA, Schindler E (1997) CNS involvement in AIDS: spectrum of CT and MR findings. Eur Radiol 7(7):1091–1097 9265682 10.1007/s003300050260

[66] van der Meulen M, Dinmohamed AG, Visser O, Doorduijn JK, Bromberg JEC (2017) Improved survival in primary central nervous system lymphoma up to age 70 only: a population-based study on incidence, primary treatment and survival in the Netherlands, 1989–2015. Leukemia 31(8):1822–1825 28450731 10.1038/leu.2017.128

[67] van der Meulen M, Bromberg JEC, Nijland M, Visser O, Doorduijn JK, Dinmohamed AG (2021) Primary therapy and survival in patients aged over 70-years-old with primary central nervous system lymphoma: a contemporary, nationwide, population-based study in the Netherlands. Haematologica 106(2):597–600 32241841 10.3324/haematol.2020.247536 PMC7849552

[68] van der Meulen M, Postma AA, Smits M, Bakunina K, Minnema MC, Seute T et al (2021) Extent of radiological response does not reflect survival in primary central nervous system lymphoma. Neurooncol Adv 3(1):vdab007 33615224 10.1093/noajnl/vdab007 PMC7883767

[69] van der Meulen M, Dirven L, Bakunina K, van den Bent MJ, Issa S, Doorduijn JK et al (2021) MMSE is an independent prognostic factor for survival in primary central nervous system lymphoma. J Neurooncol 152(2):357–362 33611761 10.1007/s11060-021-03708-8 PMC7997829

[70] Villano JL, Koshy M, Shaikh H, Dolecek TA, McCarthy BJ (2011) Age, gender, and racial differences in incidence and survival in primary CNS lymphoma. Br J Cancer 105(9):1414–1418 21915121 10.1038/bjc.2011.357 PMC3241537

[71] Wassenberg MW, Bromberg JE, Witkamp TD, Terhaard CH, Taphoorn MJ (2001) White matter lesions and encephalopathy in patients treated for primary central nervous system lymphoma. J Neurooncol 52(1):73–80 11451205 10.1023/a:1010676807228

[72] Weller M (2014) Primary central nervous system lymphoma in the elderly. Oncol Res Treat 37(7–8):376–377 25138296 10.1159/000365408

[73] Weller M, Martus P, Roth P, Thiel E, Korfel A (2012) Surgery for primary CNS lymphoma? Challenging a paradigm. Neuro Oncol 14(12):1481–1484 22984018 10.1093/neuonc/nos159 PMC3499010

[74] Yamagishi Y, Sasaki N, Nakano Y, Matushita Y, Omura T, Shimizu S et al (2021) Liquid biopsy of cerebrospinal fluid for MYD88 L265P mutation is useful for diagnosis of central nervous system lymphoma. Cancer Sci 112(11):4702–4710 34523186 10.1111/cas.15133 PMC8586690

[75] Yamanaka R, Morii K, Shinbo Y, Sano M, Homma J, Tsuchiya N et al (2017) Late relapse of primary central nervous system lymphoma. Leuk Lymphoma 58(2):475–477 27397141 10.1080/10428194.2016.1201570

[76] Yu J, Du H, Ye X, Zhang L, Xiao H (2021) High-dose methotrexate-based regimens and post-remission consolidation for treatment of newly diagnosed primary CNS lymphoma: meta-analysis of clinical trials. Sci Rep 11(1):2125 33483528 10.1038/s41598-020-80724-0 PMC7822904

